# Population Structure and Antimicrobial Resistance Profiles of *Streptococcus suis* Serotype 2 Sequence Type 25 Strains

**DOI:** 10.1371/journal.pone.0150908

**Published:** 2016-03-08

**Authors:** Taryn B. T. Athey, Sarah Teatero, Daisuke Takamatsu, Jessica Wasserscheid, Ken Dewar, Marcelo Gottschalk, Nahuel Fittipaldi

**Affiliations:** 1 Public Health Ontario, Toronto, ON, Canada; 2 Bacterial and Parasitic Diseases Research Division, National Institute of Animal Health, National Agriculture and Food Research Organization, Tsukuba, Japan; 3 The United Graduate School of Veterinary Science, Gifu University, Gifu, Japan; 4 Department of Human Genetics, McGill University and Génome Québec Innovation Centre, Montreal, QC, Canada; 5 Groupe de Recherche sur les Maladies Infectieuses du Porc, Faculté de Médecine Vétérinaire, Université de Montréal, St-Hyacinthe, QC, Canada; 6 Department of Laboratory Medicine and Pathobiology, Faculty of Medicine, University of Toronto, Toronto, ON, Canada; Centers for Disease Control & Prevention, UNITED STATES

## Abstract

Strains of serotype 2 *Streptococcus suis* are responsible for swine and human infections. Different serotype 2 genetic backgrounds have been defined using multilocus sequence typing (MLST). However, little is known about the genetic diversity within each MLST sequence type (ST). Here, we used whole-genome sequencing to test the hypothesis that *S*. *suis* serotype 2 strains of the ST25 lineage are genetically heterogeneous. We evaluated 51 serotype 2 ST25 *S*. *suis* strains isolated from diseased pigs and humans in Canada, the United States of America, and Thailand. Whole-genome sequencing revealed numerous large-scale rearrangements in the ST25 genome, compared to the genomes of ST1 and ST28 *S*. *suis* strains, which result, among other changes, in disruption of a pilus island locus. We report that recombination and lateral gene transfer contribute to ST25 genetic diversity. Phylogenetic analysis identified two main and distinct Thai and North American clades grouping most strains investigated. These clades also possessed distinct patterns of antimicrobial resistance genes, which correlated with acquisition of different integrative and conjugative elements (ICEs). Some of these ICEs were found to be integrated at a recombination hot spot, previously identified as the site of integration of the 89K pathogenicity island in serotype 2 ST7 *S*. *suis* strains. Our results highlight the limitations of MLST for phylogenetic analysis of *S*. *suis*, and the importance of lateral gene transfer and recombination as drivers of diversity in this swine pathogen and zoonotic agent.

## Introduction

Infections leading to sepsis, meningitis and other diseases in swine caused by *Streptococcus suis* often translate into significant economic losses to the porcine industry [1]. *S*. *suis* is also a zoonotic pathogen affecting individuals working in swine production or those who consume raw or undercooked pork. Severe diseases caused by this pathogen in humans include meningitis and toxic shock-like syndrome [2]. Outbreaks in China have affected hundreds of people, and led to more than forty deaths [3]. *S*. *suis* has also been identified as one of the leading causes of adult bacterial meningitis in Vietnam and Thailand [4, 5]. However, large outbreaks of *S*. *suis* disease in humans have not been described in Europe or North America [6], where many fewer cases of *S*. *suis* human disease have been reported [6].

The primary typing method to classify *S*. *suis* is based on a serological reaction against the polysaccharide capsule [1]. Decades of investigation have shown that among the 35 serotypes that have been described, strains of serotype 2 are the most frequently isolated from cases of both swine and human disease [7]. However, in North America, serotype 2 *S*. *suis* strains are less frequently recovered from diseased pigs than in other parts of the world [8]. Serotype 2 *S*. *suis* strains can be divided into at least 16 different sequence types (STs) using a multilocus sequence typing (MLST) scheme [7, 9]. ST1 strains are most commonly found in Europe and Asia, while in North America, ST25 and ST28 strains are more prevalent [7, 10]. Previous work using experimental infection of animals has shown that ST1 strains are significantly more virulent than ST28 strains, while ST25 strains showed an intermediate virulence [11, 12]. Circularized, complete genome sequences of >10 serotype 2 *S*. *suis* of various STs are now available, but, with a few exceptions, the intra-ST population structure of many relevant *S*. *suis* STs remains poorly known. Recently, we sequenced the genomes of a collection of ST28 serotype 2 strains and discovered an unexpected complex population structure and range of virulence differences among ST28 strains, a group of organisms that were previously considered to be genetically highly homogeneous [12]. However, the intra-ST25 variation has not yet been investigated, despite the fact that ST25 strains appear to be more virulent and have been associated with human disease in different continents [13, 14].

In addition to defining the *S*. *suis* population structure, the increase in genomic research is helping define the extent of the *S*. *suis* resistome [15]. For example, genes encoding resistance against tetracycline, macrolides, aminoglycosides, chloramphenicol, and other antimicrobial drugs have been identified in the various sequenced *S*. *suis* genomes [16, 17]. As in other streptococci [18], many of the resistance genes identified in *S*. *suis* have been found to be carried by integrative and conjugative elements (ICEs), transposons, genomic islands, phages, and chimeric elements [15]. However, little is known about antimicrobial resistance among ST25 strains.

Here, we sequenced the genomes of 51 serotype 2 ST25 *S*. *suis* strains isolated in three different countries (Canada, United States of America and Thailand). We used the genomic data to evaluate the ST25 population structure and define antimicrobial resistance gene content, which we linked to phenotypic antimicrobial data. We report genetic diversity associated with geographical origin among ST25 strains. We also describe differences among strains in antimicrobial resistance, and mobile genetic element content.

## Materials and Methods

### Strains, culture conditions and DNA preparation

We used a strain collection comprising 51 serotype 2 ST25 *S*. *suis* isolates (40 from Canada, 3 from the United States of America, and 8 from Thailand, S1 Table). These strains were all available ST25 *S*. *suis* isolates that we could confidently identify in our laboratory collection. Isolates from Thailand were collected between 2000 and 2002, while isolates from North America were for the most part collected from 2005 to 2011 (S1 Table). Thus, isolates from Thailand and North America are not temporally matched. Forty-one isolates were recovered from diseased pigs in non-related farms; the additional 10 isolates were from human cases (S1 Table). Serotyping and MLST had been performed previously using standard procedures [9, 19]. Strains were cultured at 37°C with 5% CO_2_ on Columbia blood agar plates containing 5% sheep blood. Liquid cultures were grown in Todd-Hewitt broth supplemented with 0.2% yeast extract. DNA was prepared from 5 ml of overnight cultures using the QIAamp DNA minikit (Qiagen, Toronto, ON, Canada) following the manufacturers’ protocol for Gram positive organisms.

### Whole-genome sequencing and closure of a reference ST25 genome

Whole-genome sequencing libraries were prepared for all 51 isolates using Nextera XT kits (Illumina, San Diego, CA, USA) and sequenced as paired-end with either HiSeq 2500 (101 bp + 101 bp) or MiSeq (150 bp + 150 bp) instruments (Illumina). Parsing of the multiplexed sequencing reads and removal of barcode information was performed using onboard software. Short-read sequences have been deposited in NCBI’s Sequence Read Archive (Accession numbers are provided in S1 Table). MLST of all isolates was confirmed from short-read whole genome sequencing data using SRST2 software [20]. We also sequenced to closure the genome of strain NSUI060 using SMRT sequencing (Pacific Biosciences, Menlo Park, CA, USA). This strains was previously designated 1085543 and its growth and virulence characteristics have been studied extensively [11]. Briefly, two SMRT cells of sequence were run using P4C2 reagents and protocols, generating 54,923 reads exceeding 3kb in length (average read length of 6.1 kb; 146X coverage for reads >3 kb). We used HGAP v2 [21] to correct the long reads and Celera Assembler 7.0 [22] to assemble the corrected reads, followed by two rounds of polishing with Quiver (https://github.com/PacificBiosciences/GenomicConsensus). To assess base-calling accuracy in the assembly, Illumina short-reads were aligned to the assembly using BLAT [23]. The genome assembly was completely concordant with full-length perfectly aligning Illumina short-reads. The genome was formatted to begin at the first nucleotide of the intergenic region immediately preceding gene *dnaA*, encoding a chromosomal replication initiation protein. The finalized genome was annotated using Prokka [24] and deposited in GenBank under Accession number CP012911. Genome illustrations were generated with BRIG [25].

### Phylogenetic analysis, core-genome, and assessment of recombination

Single-nucleotide polymorphisms (SNPs) relative to the genome of the ST25 reference strain NSUI060 were identified for each of the 50 additional ST25 strains using VAAL [26]. SNPs were sorted and assessed using custom scripts as previously described [12]. Sliding window plots of SNP positions were created for each strain in R using custom scripts and a window of 5 kbp [27]. The A5 pipeline was used for *de novo* assembly of Illumina sequenced strains [28]. Contigs were ordered relative to the NSUI060 reference genome using Progressive Mauve [29]. Then, pseudochromosomes were created for the remaining 50 strains by concatenating the ordered contigs using the sequence NNNNNCATTCCATTCATTAATTAATTAATGAATGAATGNNNNN, which introduces start and stop codons in all 6 reading frames, as a separator. *De novo* assembled pseudogenomes were annotated using Prokka [24]. We identified orthologues using Inparanoid [30] and Quickparanoid (http://pl.postech.ac.kr/QuickParanoid). A gene was considered to be part of the ST25 core genome if it was present in all strains, and it differed in length by no more than 9 bp among all strains. For recombination analysis, core genome genes were aligned using Muscle v3.7 [31], and gaps were removed using trimAl v1.2 [32]. These aligned and trimmed genes were then reassembled for each strain in the order in which the gene appeared in the NSUI060 reference genome. Recombination occurring in the so defined core genome was assessed using BRATNextGen [33], run with 20 iterations and 100 replications, using a p-value of 0.05 as the significance cut-off. Neighbor joining trees (1,000 bootstrap replications) were created using Splitstree [34].

### Antimicrobial resistance, antimicrobial resistance gene and mobile genetic element content

MICs for penicillin, ampicillin, tetracycline, erythromycin, florfenicol, ceftiofur, and enrofloxacin were determined by using the agar dilution method [35]. The presence of antibiotic resistance genes was determined from whole-genome short-read data using SRST2 and its antibiotic resistance database [20]. ICEs were identified by manual inspection of the NSUI060 genome, specifically by searching for integrases and other known ICE related genes [36] and comparing the NSUI060 surrounding genomic content with those of serotype 2 ST1 strain P1/7, and serotype 2 ST28 strain NSUI002 to determine ICE boundaries. Then, alignment of short-read data of all strains to the regions of the NSUI060 genome using Mosaik [37] permitted us to identify which of these strains possessed the same mobile genetic elements (MGEs). We next scanned *de novo* assemblies of all other strains to identify additional MGEs. Comparison of ICEs was performed with Easyfig [38].

## Results and Discussion

### Genome comparisons identify large genome rearrangements in a serotype 2 ST25 *S. suis* strain

**Fig 1:**
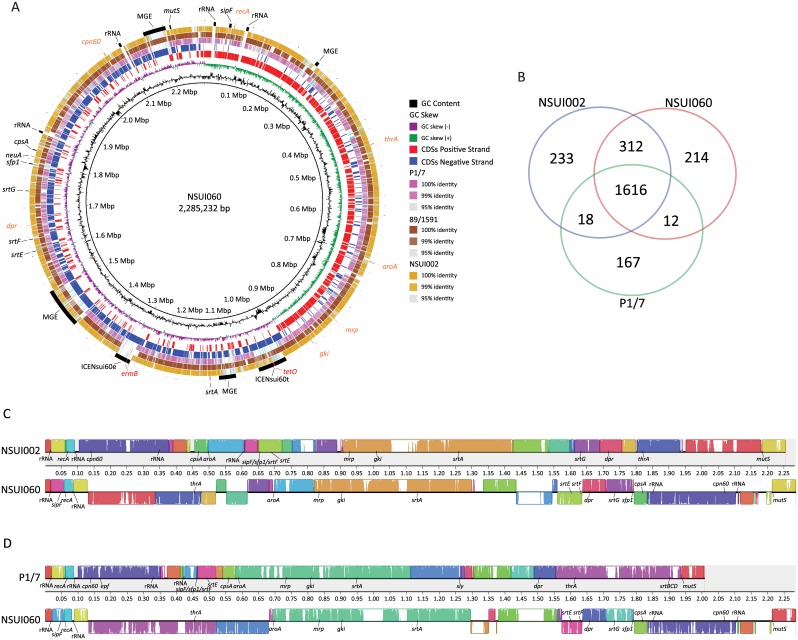
A) Genome atlas of Canadian *S*. *suis* ST25 strain NSUI060. Depicted data from innermost to outer most circles represent genome size in Mbp (circle 1); percent G+C content (circle 2); GC skew (G—C)/(G + C) averaged over a moving window of 10,000 bp, with excess G and excess C shown in green and purple, respectively (circle 3); annotated coding sequences (CDSs) encoded on the forward/direct (circle 4, red), and reverse/complementary (circle 5, blue) chromosomal strands; TBLASTN comparisons of the CDSs predicted in ST25 strain NSUI060 and ST1 strain P1/7 (circle 6; percent identity defined in the body of the figure); TBLASTN comparisons of the CDSs predicted in ST25 strains NSUI060 and 89/1591 (circle 7; percent identity defined in the body of the figure); TBLASTN comparisons of the CDSs predicted in ST25 strain NSUI060 and ST28 strain NSUI002 (circle 8; percent identity defined in the body of the figure); reference genome landmarks (circle 9): ribosomal RNAs, capsule and pilus related genes, and mobile genetic elements are labelled in black, genes used in the *S*. *suis* MLST scheme are labelled in orange; genes encoding resistance to antimicrobial agents are labelled in red. MGE: Mobile genetic element. B) Venn diagram depicting unique and shared gene clusters in *S*. *suis* strains as identified by ortholog analysis. The ST28 strain NSUI002 is represented by the blue circle, the ST25 strain NSUI060 is represented by the red circle, and the ST1 strain P1/7 is represented by the green circle. Numbers in the intersectional regions indicate gene clusters shared by strains. Colinearity of the genomes of *S*. *suis* ST25 strain NSUI060 and C) ST28 strain NSUI002 or D) ST1 strain P1/7. The genomes of the strains were aligned using progressiveMauve. Sequence alignments that are free of rearrangements are shown as colored local collinear blocks (LCBs). Sequence inversions are denoted by differential positioning of LCBs relative to a reference axis. Pilus related genes, rRNAs, genes used in MLST typing are labelled and other landmarks are indicated.

To facilitate downstream bioinformatics analysis we first sequenced to closure the genome of Canadian ST25 strain NSUI060. The genome was a circular chromosome of 2,285,232 bp (Fig 1A) with a G+C content of 41.1%. The strain also contained two plasmids, pNSUI060a and pNSUI060b, of 11,393 and 5,581 bp, respectively (S1 Fig). We identified 2,324 CDSs in the NSUI060 chromosome. Of them, 352 corresponded to genes found in 6 MGEs (Fig 1A). Two of these MGEs (ICENsui60t and ICENsui60e) carried genes *tetO* and *ermB*, conferring resistance to tetracyclines and macrolides, respectively. The draft genome of a different *S*. *suis* serotype 2 ST25 strain is available in GenBank (strain 89/1591, Accession Number GCA_000167375.1). Aligning of available sequences of strain 89/1591 to the newly sequenced genome identified a high level of global homology (Fig 1A). We next compared the genome of NSUI060 to that of the recently released genome of serotype 2 ST28 strain NSUI002 [12] (GenBank Accession Number CP011419), and to the genome of the highly virulent serotype 2 ST1 strain P1/7 (GenBank Accession Number GCA_000091905) [16]. Previous work has shown that both ST25 and ST28 strains are less virulent than ST1 strains in the mouse and porcine models of infection [11, 39]. Consistent with the previous hypothesis that more virulent *S*. *suis* strains have smaller genome sizes than their less virulent counterparts [40, 41], the size of the ST25 and ST28 genomes were much larger than that of the ST1 strain (S2 Table). These differences in genome size correlated with the abundance of MGEs in the genomes of the ST25 and ST28 strains. We also identified that ST25 strain NSUI060 and ST28 strain NSUI002 shared much more gene content with each other than either of them shared with ST1 strain P1/7 (Fig 1B). Further inspection discovered that approximately 85% of genes unique to ST25 and approximately 85% of genes unique to ST28 were found in MGEs. As well, approximately 65% of the genes shared between the ST25 and ST28 strains but not found in the ST1 strain corresponded to gene content present in MGEs.

The genome of the ST25 strain did not contain genes *sly* and *epf* (encoding the hemolysin known as suilysin, and an extracellular factor, also referred to as EF, respectively). These genes are also absent from the ST28 genome, but both are found in the genome of ST1 strain P1/7 [42–44]. On the other hand, we identified a copy of *mrp* gene variant *** [45] in the genome of the ST25 strain NSUI060. However, the *mrp* gene had a nonsense mutation at the 5’ end predicted to result in a premature stop codon. Consistently, MRP was not detected by immunoblot in either the supernatant or the cell-wall fraction of NSUI060 (data not shown). Both the ST25 and ST28 strains possessed a *srtG* pilus island [46], which was absent from the ST1 strain P1/7, but neither possessed the *srtBCD* pilus cluster, found in P1/7.

One unexpected finding of our genome comparisons was that the genome of the ST25 presents 27 genome rearrangements compared to the genomes of ST28 strain NSUI002 and ST1 strain P1/7 (Fig 1C and 1D). One of these inversions results in truncation of the so-called *srtF* pilus island, whose 4 genes are separated by > 400 Kbp in the genome NSUI060 (Fig 1A). Thus, our results provide an explanation to the intriguing observation that ST25 strains do not express the *srtF* pilus, even if all *srtF* pilus island genes were found to be present in ST25 strains using a previously described PCR pilus typing scheme [11, 46]. We also discovered that the *cps* locus, whose genes encode proteins catalyzing biosynthesis of the polysaccharide capsule, and which is found upstream of gene *aroA* in both ST28 and ST1 strains, is located 1.2 Mbp downstream of *aroA* in the ST25 genome (Fig 1A). Although their biological role is poorly understood, inversions in the genomes of streptococci such as Group A *Streptococcus* and *Streptococcus mutans* are not unusual [47, 48]. Symmetrical genome inversions in bacterial organisms have been suggested to correlate with enhanced bacterial fitness as they occur most frequently in clinical isolates [49, 50]. However, inversions found in NSUI060 appear to be asymmetrical and linked to acquisition of MGEs, as shown by the fact that >85% of inversion boundaries contain genes encoding for transposases and/or integrases. Another interesting observation is that as a result of genome inversions, the distribution of rRNA operons throughout the genome is different in the ST25 strain than in other serotype 2 *S*. *suis* genomes (Fig 1C and 1D).

### Recombination and population structure of serotype 2 ST25 *S*. *suis* strains

**Fig 2:**
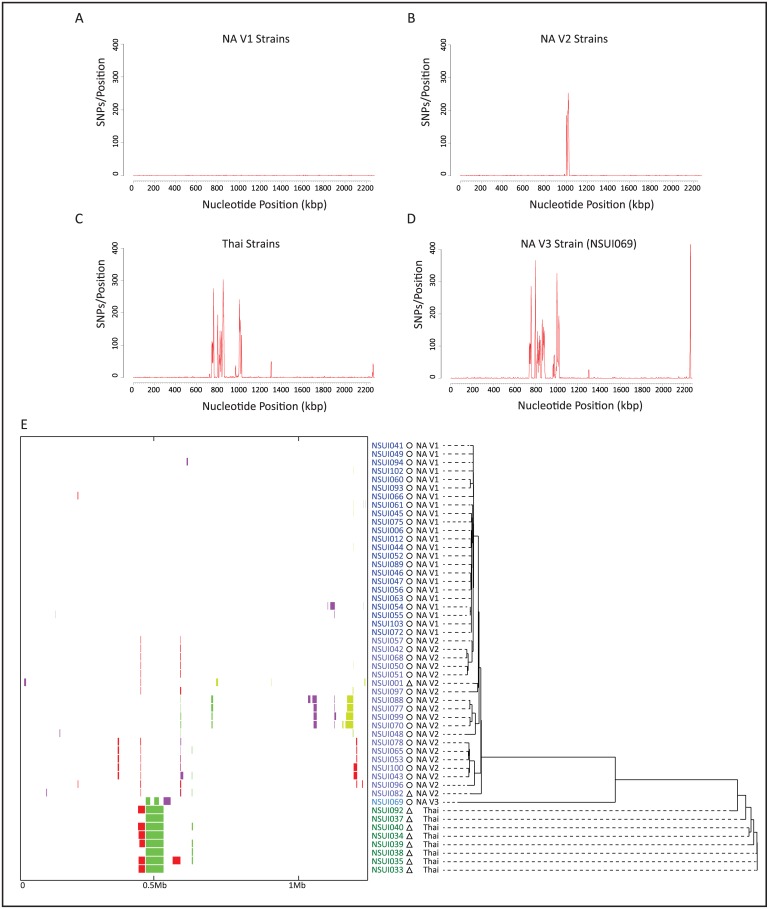
Sliding window analysis of single-nucleotide polymorphism (SNP) distribution, recombination analysis and phylogenetic relationships among *S*. *suis* ST25 strains. **A)** In a group of 23 North American strains (defined as North American variant 1, NA V1 Strains) SNPs were distributed across the genome of the reference strain. Sliding windows were created using R-software and custom scripts and a window of 5 kbp. **B)** In a group of 19 North American strains (NA V2 Strains), SNPS distribution was non-random, and an overabundance of polymorphisms was identified between positions 1 to 1.035 Mbp of the NSUI060 genome. **C)** In all 8 Thai strains, SNP distribution was non-random, with two distinct peaks (positions ~740 kbp to ~760 kbp and ~790 kbp to ~860 kbp of the reference genome) showing overabundance of SNPs. **D)** SNP distribution in a single Canadian strain (NSUI069) also identified overabundance of SNPs in discrete areas of the reference genome. **E)** The left panel depicts results of Bayesian analysis of recombination for 51 ST25 *S*. *suis* strains. The colored bars denote recombination events identified for each strain relative to the core ST25 genome. The coloring of the bars at a specific genomic location reflects the clustering of the recombination events into groups, and is unrelated to other bars at distant genomic locations. The right panel shows a Neighbor-joining phylogenetic tree constructed using 3,023 non redundant SNP loci identified among all ST25 *S*. *suis* strains relative to the core ST25 genome devoid of areas having undergone recombination. The different clades are indicated by the different colors used to label strain names, circles represent strains that were isolated from a swine host, and triangles represent strains that were isolated from a human host. Variants defined by sliding window analysis are also indicated.

One limitation of MLST is that this typing scheme only uses a minimal fraction of the *S*. *suis* genome (7 genes) to identify genetic diversity. Thus, MLST may group together strains that present extensive genomic variation, as we recently demonstrated using whole-genome sequencing analysis of ST28 serotype 2 *S*. *suis* [12]. The intra-ST25 population structure is poorly known. In particular, it is not known whether ST25 strains are genetically closely related, or whether, like ST28 strains, they constitute a heterogeneous group of organisms. To begin to address these and other questions, we used genomics and a convenience sample of 50 additional ST25 serotype 2 strains isolated in Canada, the United States of America and Thailand, countries where ST25 strains are frequently isolated [11, 14]. A total of 8,356 non-redundant SNPs were identified among all strains relative to the NSUI060 reference genome. The average number of SNPs differentiating ST25 strains between themselves was 533. Preliminary inspection showed that these SNPs were not randomly distributed across the genome in some strains. Instead, several discrete areas of the genome had an overabundance of polymorphisms, suggesting recombination. To investigate the issue in more detail, we performed sliding window analysis. We identified four distinct profiles (Fig 2A–2D). In the first group, comprising 23 strains from North America (identified as NA V1 strains, Fig 2A), the polymorphisms appear to be evenly distributed across the genome of the reference strain. A second North American group (identified as NA V2 strains, Fig 2B) comprised 19 strains. In these strains, we identified overabundance of SNPs between positions ~1 to ~1.035 Mbp of the NSUI060 genome. Inspection of the genome annotation at those positions revealed the presence of an MGE in NSUI060. A third group was formed by all Thai strains, whose polymorphism distribution presented two distinct peaks from ~740 kbp to ~760 kbp and from ~790 kbp to ~860 kbp, in addition to the abovementioned peak at ~1 Mbp (Fig 2C). We finally identified a single isolate (strain NSUI069, from Canada) which had a unique profile of SNP distribution (NA V3, Fig 2D).

To more precisely study recombination among ST25 strains, we eliminated from the analysis all genome regions identified to be MGEs, and used ortholog analysis to define a core-ST25 genome. Core genes for each strain were concatenated in the order they appeared in the NSUI060 reference, and areas of recombination between strains were defined using BRATNextGen [33]. The analysis revealed large areas of recombination differentiating Thai and North American strains (Fig 2E, left panel), corresponding to two distinct peaks identified by sliding windows analysis. The first peak region (~740 kbp to ~760 kbp of the reference genome) contains the *dlt* operon, involved in modifications of cell-wall techoic acid by D-alanylation, which has been shown to be important for *S*. *suis* virulence [51]. The second highly variable region (~790 kbp to ~860 kbp of the reference genome) contains the gene *mrp*. We identified that ST25 strains from Thailand and ST25 strains from North America possess different variants of the gene (variants mrp**, and mrp***, respectively). The North American and the Thai *mrp* alleles have 97% DNA sequence identity. Interestingly, the North American singleton NSUI069 had a third *mrp* allele (*mrp*^*s*^), which had an average of 86% DNA sequence identity to the mrp**, and mrp*** variants identified in Thai and North America strains. Despite these differences, we identified that gene *mrp* was interrupted in all North American ST25 strains by different point mutations and/or small insertion/deletions occurring at the 5’ end of *mrp*, resulting in premature termination of translation. Thus, none of these ST25 is expected to express MRP. Recombination among North American strains was less important and involved 11.6% of the core genome (Fig 2E). This value is substantially lower than the percentage of genomic recombination that was determined for ST28 strains from North America (26.5%) [12]. A similar lower level of recombination was identified between the Thai strains when they were considered as a group.

We next established phylogenetic relationships between the ST25 strains using 3,023 SNPs relative to the core genome of strains NSUI060 that remained after areas of recombination were eliminated from the analysis. Essentially, 2 different clades (Fig 2E, right panel and S2 Fig) were identified. A first clade comprises the vast majority of the North American strains, including recombination variants NA V1 and NA V2. Diversity within NA V1 strains was minimal, while NA V2 strains could be differentiated in several subclades. A second clade is formed by all Thai strains. The genetic differences between the Thai strains were also limited. While the two main clades show a strong signal of geographical structure suggesting that they constitute two differentiated ST25 subpopulations, it must be noted that members of the two clades are also temporally unrelated. Indeed, strains from Thailand were isolated in years 2000 to 2002, while North American strains were recovered from 2005 to 2011. Thus, temporal differences in addition to geography may be influencing the observed clustering. The Thai clade is formed by ST25 isolates recovered from human infections only. Because our collection did not contain temporally matched porcine isolates from Thailand it is difficult to support the speculation that the Thai clade constitutes a “human-specific” ST25 subpopulation. Actually, we consider this hypothesis unlikely, based on the fact that the overwhelming majority of human *S*. *suis* cases reported in the literature have been linked to concurrent porcine *S*. *suis* outbreaks, close contact with porcine byproducts, or consumption of pork [6]. In addition, analysis of our data showed that in North America, strains isolated from both human and swine are genetically closely related (Fig 2E).

We also identified one singleton (strain NSUI069), which was separated by a similar genetic distance from the bulk of North American and Thai strains (Fig 2E, labeled in light blue). Interestingly, we had identified that strain NSUI069 had a unique recombination pattern, involving, roughly, positions 850kb to 900kb of the genome of the reference strain NSUI060. This region contains many regulatory genes, such as *carAB* (encoding carbamoyl phosphate synthases involved in glutamine synthesis), a gene encoding a Fic family protein (involved in cell division), and genes encoding spermidine/putrescine ABC transporter systems [52, 53]. Recombination analysis allowed us to define a more stringent core genome and offered an explanation to large areas of difference between North American strains, Thai strains, and the outlier strain NSUI069.

### Antimicrobial resistance in *S*. *suis* ST25 strains

We next determined susceptibility of the ST25 strains to a battery of antimicrobials commonly used to treat infections in swine. All strains were susceptible to penicillin, ampicillin, and ceftiofur (Table 1). All strains had intermediate (i.e., reduced) susceptibility to florfenicol. Accordingly to CLSI guidelines for *S*. *suis*, 15 strains (29%) had intermediate susceptibility to enrofloxacin, while one strain (2%) was resistant. The remainder of the strains (35/51, 69%) were susceptible to this antimicrobial (Table 1). Enrofloxacin resistance is associated with point mutations in the quinolone-resistance determining regions (QRDR) of the genes *gyrA* and *parC* and/or with the action of specific efflux pumps [54]. However, analysis of these gene regions in our intermediate or resistant strains did not find SNPs associated with fluoroquinolone resistance (S3 and S4 Figs). We also could not identify efflux pumps associated with fluoroquinolone resistance in the genomes of the ST25 strains (data not shown). Thus, we are unable to provide details about the mechanisms leading to reduced susceptibility to enrofloxacin among the strains in our collection. However, as stated above, we used the CLSI guidelines to establish the enrofloxacin resistance status of our strains. Accordingly to these guidelines, strains with MIC ≤ 0.5 μg/mL are susceptible, those with MICs of 1 μg/mL are intermediate, and strains with MICs ≥ 2 μg/mL are resistant. However, these values are inconsistent with many studies assessing how mutations in *gyrA* and *parC* confer antimicrobial resistance to fluoroquinolones. Based on the literature, we believe that CLSI guidelines may not adequately assess resistance in *S*. *suis*. For example, Rui *et al*. found that a single amino acid change in the QRDR regions of *gyrA* and *parC* increased the MIC from 0.5 μg/mL to 8 μg/mL [55]. Similarly, when Yao *et al*. introduced a single mutation into a susceptible *S*. *suis* strain, the MIC for this strain increased from 0.5 μg/mL to 128 μg/mL [56]. Alternatively, Escudero *et al*. described a strain that contained a single mutation in *parC* but an MIC of only 1μg/mL. This strain, however, was the only strain in that study to contain this specific mutation, and though it contained a low MIC to enrofloxacin, it was highly resistant to norfloxacin (MIC 32 μg/mL) [54]. As well, several studies have reported strains with enrofloxacin MICs of ≤ 1 μg/mL as being susceptible to this antimicrobial [56–58]. Readers should bear in mind these studies when evaluating enrofloxacin results reported here.

**Table 1:** Antimicrobial susceptibility of ST25 *S*. *suis* strains used in this study.

Antimicrobial agent	N° of susceptible strains^a^ (%)	N° of strains with intermediate resistance^b^ (%)	N° of resistant strains^c^ (%)
Ampicillin	51 (100)	0 (0)	0 (0)
Ceftiofur	51 (100)	0 (0)	0 (0)
Florfenicol	0 (0)	51 (100)	0 (0)
Enrofloxacin	35 (69)	15 (29)	1 (2)
Tetracycline	1 (2)	1 (2)	49 (96)
Penicillin	51 (100)	0 (0)	0 (0)
Erythromycin	1 (2)	0 (0)	50 (98)

^a^ Susceptible MICs for ampicillin, ceftiofur, florfenicol, enrofloxacin, tetracyline, penicillin, and erythromycin are ≤0.5, ≤2, ≤2, ≤0.5, ≤0.5, ≤0.06, and ≤0.25 μg/mL, respectively.

^b^ Intermediate MICS for ampicillin, ceftiofur, florfenicol, enrofloxacin, tetracyline, penicillin, and erythromycin are 1, 4, 4, 1, 1, 0.12–1, and 0.5 μg/mL, respectively.

^c^ Resistance MICs for ampicillin, ceftiofur, florfenicol, enrofloxacin, tetracyline, penicillin, and erythromycin are ≥2, ≥8, ≥8, ≥2, ≥2, ≥2, and ≥1 μg/mL, respectively.

**Fig 3:**
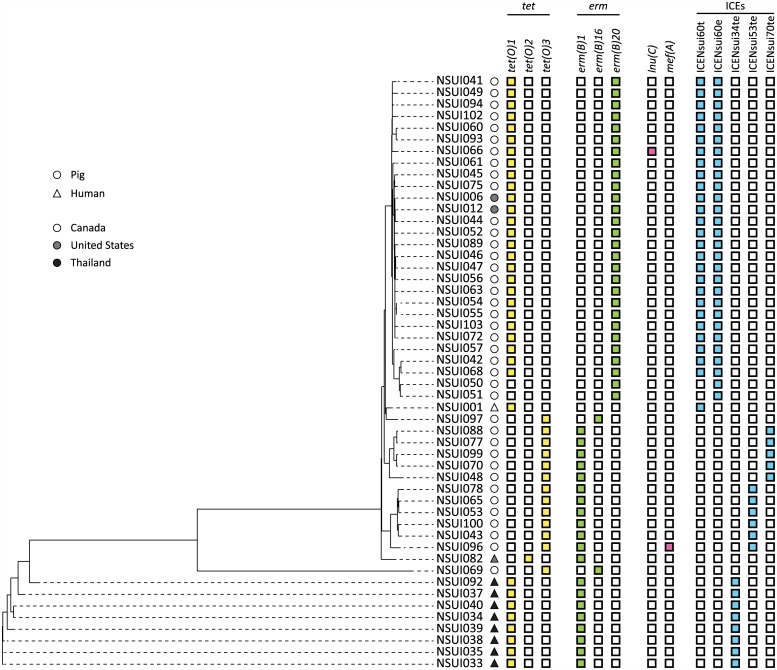
Presence of ICEs and antimicrobial resistance encoding genes in the 51 ST25 *S*. *suis* genomes. The left panel shows inferred genetic relationships between strains, determined as described in Fig 2. The right panel depicts host, country of origin, presence or absence of ICEs and/or resistance genes in the genomes of the strains under investigation. Strains isolated from pigs are depicted by a circle, while strains isolated from humans are depicted by a triangle. Strains isolated in Canada are shown as a white circle or triangle, strains isolated in the United States of America are shown in grey, and strains isolated in Thailand are shown in black. ICEs are depicted in light blue, tetracycline resistance genes are depicted in yellow, erythromycin resistance genes are depicted in light green, all other antimicrobial resistance genes are depicted in pink.

Fifty strains were resistant to erythromycin, while 49 strains were resistant to tetracycline (Table 1). Resistance to erythromycin and tetracycline correlated with the presence of genes *ermB* and *tetO*, respectively, in the genomes of all resistant strains (Fig 3). In strain NSUI060 an ~60kbp ICE (ICENsui60t) carried the *tetO* allele *tetO-1* (GenBank Accession Number: M18896) while an ~30kbp ICE (ICENsui60e) carried *ermB* allele *ermB-20* (GenBank Accession Number: AF109075) (Fig 1A). Inspection of all other genomes identified that 28 additional strains contained ICENsui60e, while 27/51 strains contained ICENsui60t (Fig 3). Li *et al*. described an 89K pathogenicity island in the virulent serotype 2 ST7 strain 05ZYH33, which lies between a hydrolase-encoding gene and a 50S ribosomal protein L7/L12-encoding gene [59]. Interestingly, the 60K ICENsui60t found in this study in strain NSUI060 is also inserted between hydrolase and 50S ribosomal L7/L12-encoding genes. However, BLAST comparisons between the 89K ICE found in 05ZYH33 (Accession number CP000407) and ICENsui60t only found ~36Kb of homology between the elements (S5 Fig). Notably, 60% of the genes found in the 89K ICE, including the NisK/R two-component system involved in virulence, a Tn916-like element, and a lantibiotic system were not identified in ICENsui60t.

**Fig 4:**
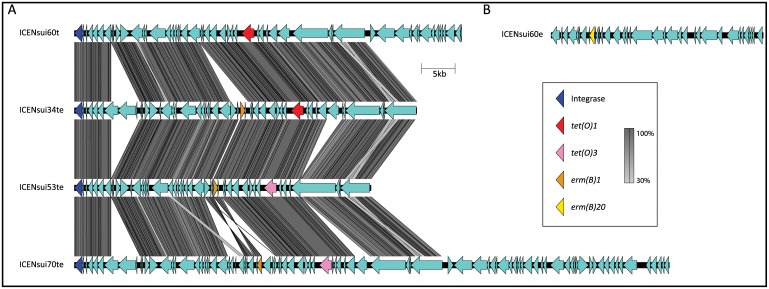
Resistance against tetracycline and macrolides is encoded by genes carried by integrative and conjugative elements (ICEs) in most ST25 *S*. *suis* strains. Genomic comparison identified at least five ICEs carrying genes encoding resistance among the 51 ST25 strains under investigation. The majority of strains, including the reference strain NSUI060 possessed two ICEs, one (ICENsui60t) carrying gene *tetO-1* (top, left), and a second (ICENsui60e) carrying gene *ermB-20* (top, right). In most other strains a single ICE carried genetic determinants for both resistances, encoded by different *tetO* and *ermB* alleles. Homology between ICE genes is depicted by the different shades of grey.

All ST25 strains devoid of ICENsui60t and ICENsui60e contained either *ermB*-*1* or *ermB-16* alleles (GenBank Accession Numbers JN899585 and X82819, respectively), in combination with *tetO-1*, *tetO-2* (GenBank Accession Number: M20925), or *tetO-3* (GenBank Accession Number: Y07780) alleles (Fig 3). These genes were carried in 3 additional ICEs (Fig 4). All Thai strains contained ICENsui34te, carrying alleles *ermB-1* and *tetO-1*. In contrast, North American strains devoid of ICENsui60t and ICENsui60e had either ICENsui53te or ICENsui070te, which both carry alleles *ermB-1* and *tetO-3*, but differ in the orientation of *ermB* and surrounding genes (Fig 4). Additionally, we were unable to identify any of these described ICEs, nor other ICES in the genomes of three strains (NSUI069, NSUI082, and NSUI097). However, two of these strains possessed allele *ermB-16* and *tetO-3*, while the third had alleles *ermB-1* and *tetO-2*.

The acquisition of resistance carrying ICEs by zoonotic pathogens is of great public health concern. Different *Streptococcus* species have been shown to have similar ICE insertion sites, which can lead to cross-species ICE integration [60]. As well, previous studies have shown that *S*. *suis* ICEs can recombine with ICEs belonging to other *Streptococcus* species, such as *Streptococcus agalactiae and Streptococcus pyogenes* [61]. Therefore, antibiotic resistance genes acquired by veterinary pathogens such as *S*. *suis* may lead to the acquisition of resistance in human specific pathogens [15].

## Concluding Remarks

Rapid and relatively inexpensive closure of bacterial pathogen genomes is now accessible to most research laboratories [62]. The added information that can be obtained holds the promise of revolutionizing our understanding of bacterial virulence and result in new leads to better understand the pathogenesis of infection. One of our key findings was that the genome of ST25 strains present several rearrangements compared to those of ST1 and ST28 strains. Notably, at least one of these rearrangements results in truncation of the *srtF* pilus island. The role of pili in *S*. *suis* virulence is controversial [63, 64]. However, pili have been considering interesting vaccine candidates. It has been shown that a large proportion of *S*. *suis* strains causing disease in pigs and humans, including those of ST25, have all required genes for *srtF* pilus biosynthesis [46]. However, we show here that genome rearrangements result in inability of ST25 strains to express a functional *srtF* pilus. Thus, pilus-based vaccine formulations may need to include other subunits such as those encoded by the *srtG* pilus island [46, 64] to provide protection against ST25 *S*. *suis* disease. Disruption of the *srtF* pilus is only one example of the potential effect of genome rearrangement in *S*. *suis*. Further investigation of the consequences of genome rearrangements in *S*. *suis* should be considered in prospective international studies.

The use of whole-genome sequencing permitted us to detect genetic differences which are invisible to the MLST scheme commonly used to characterize *S*. *suis* strains [9]. Using a similar genomics approach, we recently reported a relatively high level of genetic diversity among a large collection of serotype 2 ST28 *S*. *suis* strains isolated from diseased pigs and humans in different geographies [12]. Here, we report that the population structure of 51 serotype 2 ST25 *S*. *suis* strains is defined by comparatively less diversity. However, phylogenetic analysis identified with two clearly distinct main clades composed of Thai and North American strains, respectively. Variable gene content, notably ICEs and recombination in the core genome were also observed to correlate with geographical area of isolation of the strains (Thai vs North America). Interestingly, whole-genome-based SNP phylogenies revealed that the North American clade could be subdivided into two main subclades, which differed mainly in ICE content and had defined patterns of antimicrobial resistance genes. However, within-clade variation was observed both in terms of recombination and lateral gene transfer, as well as by numerous small genetic changes such as short indels and SNPs. We believe that further study of the consequences of these genetic changes (i.e., impact in global transcription patterns leading to potential differential expression of virulence genes) are necessary next steps to begin to elucidate at the population level the molecular underpinnings of the *S*. *suis*-host interaction.

## Supporting Information

S1 Fig Representation of genes present in NSUI060 plasmids. CDSs depicted in red are on the forward coding strand, CDSs depicted in blue are on the reverse coding strand.
(PDF)

S2 Fig Phylogenetic relationships between 51 ST25 *S*. *suis* strains. The tree was rooted using Canadian ST28 strain NSUI002 as an outgroup.
(PDF)

S3 Fig Comparison of amino acid sequence of *gyrA*. The amino acid sequence for the reference strain NSUI060 is shown at the top. Dots represent conserved regions, the scale at the top represents amino acid sequence position. MICs in μg/mL to enrofloxacin for each strain are shown to the right of the figure.
(PDF)

S4 Fig Comparison of amino acid sequence of *parC*. The amino acid sequence for the reference strain NSUI060 is shown at the top. Dots represent conserved regions, the scale at the top represents amino acid sequence position. MICs in μg/mL to enrofloxacin for each strain are shown to the right of the figure.
(PDF)

S5 Fig Comparison of integrative and conjugative elements found in 05ZYH33 and NSUI060 containing *tet* resistance conferring genes. The shades of grey depict the percent homology between elements. Integrases are depicted in dark blue, the NisK/R two-component system is depicted in green, tetrocycline resistance conferring genes are depicted in orange, lantibiotic proteins are depicted in red, and genome content is depicted in brown.
(PDF)

S1 Table *Streptococcus suis* strains used in this study. (PDF)

S2 Table Characteristics of the NSUI060 genome and previously closed NSUI002 and P1/7 *Streptoccocus suis* genomes. (PDF)
