# Supplementary material for: Population Structure and Antimicrobial Resistance Profiles of Streptococcus suis Serotype 2 Sequence Type 25 Strains
Source: PLoS One. 2016 Mar 8;11(3):e0150908. doi: 10.1371/journal.pone.0150908 (PMC4783015; doi:10.1371/journal.pone.0150908)
Supplement: S1 Fig — CDSs depicted in red are on the forward coding strand, CDSs depicted in blue are on the reverse coding strand. (PDF) [file pone.0150908.s001.pdf]

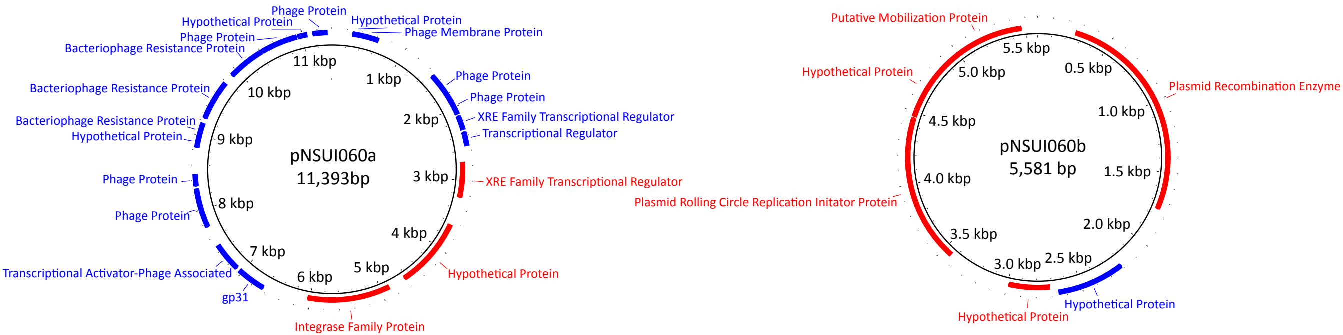

S1 Fig. Representation of genes present in NSUI060 plasmids. CDSs depicted in red are on the forward coding strand, CDSs depicted in blue are on the reverse coding strand.
