# Supplementary material for: Population Structure and Antimicrobial Resistance Profiles of Streptococcus suis Serotype 2 Sequence Type 25 Strains
Source: PLoS One. 2016 Mar 8;11(3):e0150908. doi: 10.1371/journal.pone.0150908 (PMC4783015; doi:10.1371/journal.pone.0150908)
Supplement: S2 Fig — The tree was rooted using Canadian ST28 strain NSUI002 as an outgroup. (PDF) [file pone.0150908.s002.pdf]

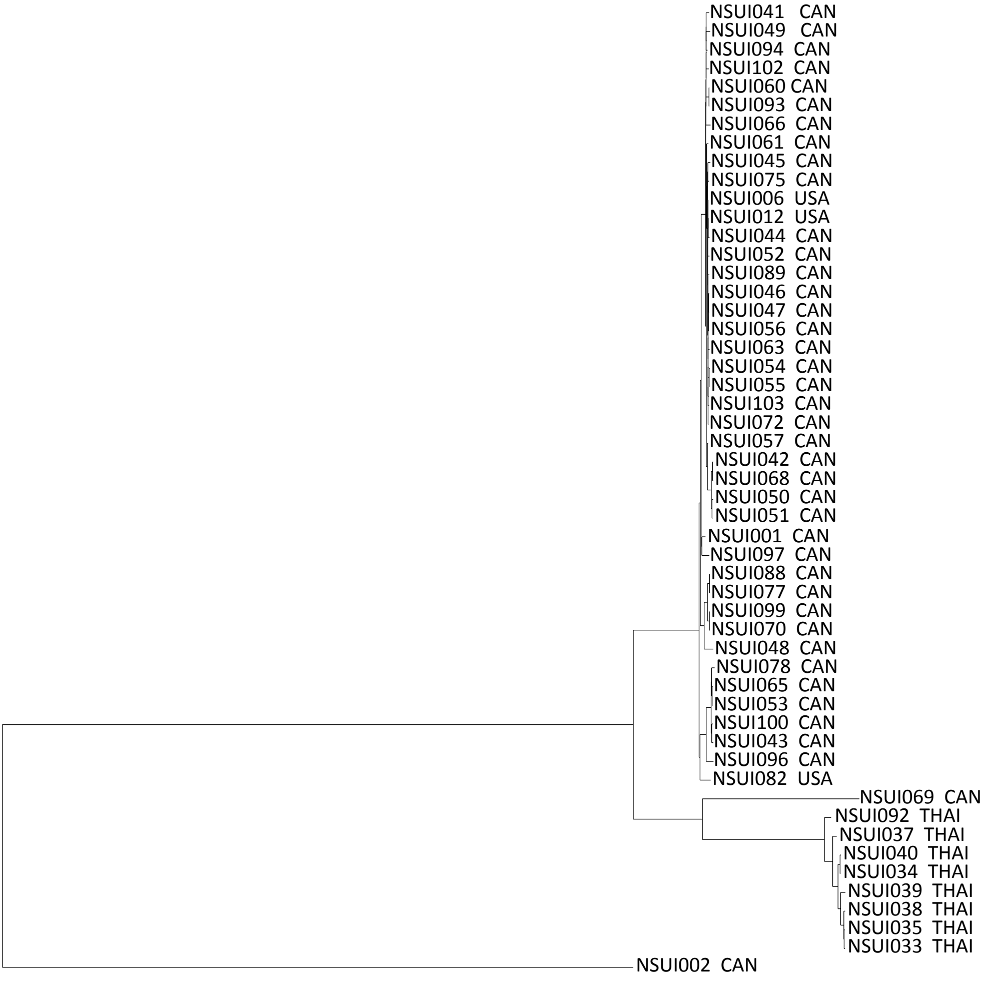

S2 Fig. Phylogenetic relationships between 51 ST25 *S. suis* strains. The tree was rooted using Canadian ST28 strain NSUI002 as an outgroup.
