# Supplementary material for: Population Structure and Antimicrobial Resistance Profiles of Streptococcus suis Serotype 2 Sequence Type 25 Strains
Source: PLoS One. 2016 Mar 8;11(3):e0150908. doi: 10.1371/journal.pone.0150908 (PMC4783015; doi:10.1371/journal.pone.0150908)
Supplement: S3 Fig — The amino acid sequence for the reference strain NSUI060 is shown at the top. Dots represent conserved regions, the scale at the top represents amino acid sequence position. MICs in μg/mL to enrofloxacin for each strain are shown to the right of the figure. (PDF) [file pone.0150908.s003.pdf]

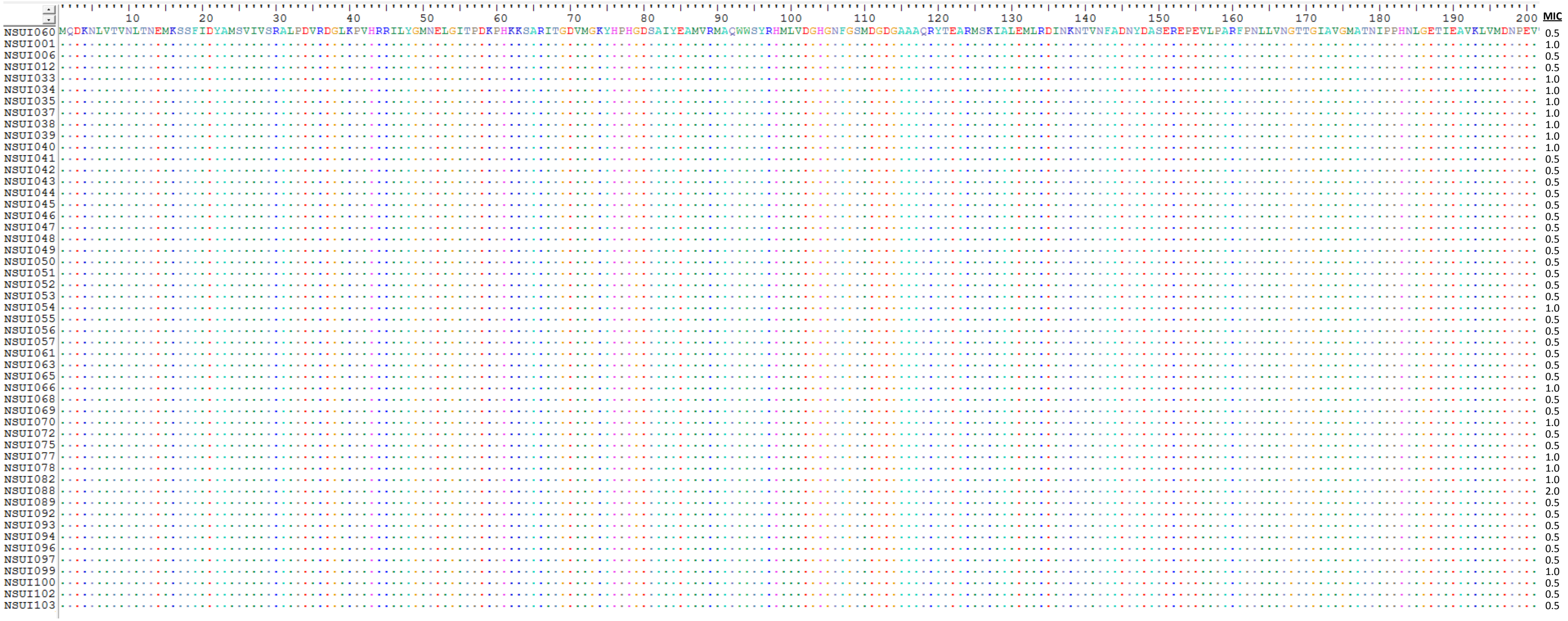

S3 Fig. Comparison of amino acid sequence of *gyrA*. The amino acid sequence for the reference strain NSUI060 is shown at the top. Dots represent conserved regions, the scale at the top represents amino acid sequence position. MICs in µg/mL to enrofloxacin for each strain are shown to the right of the figure.

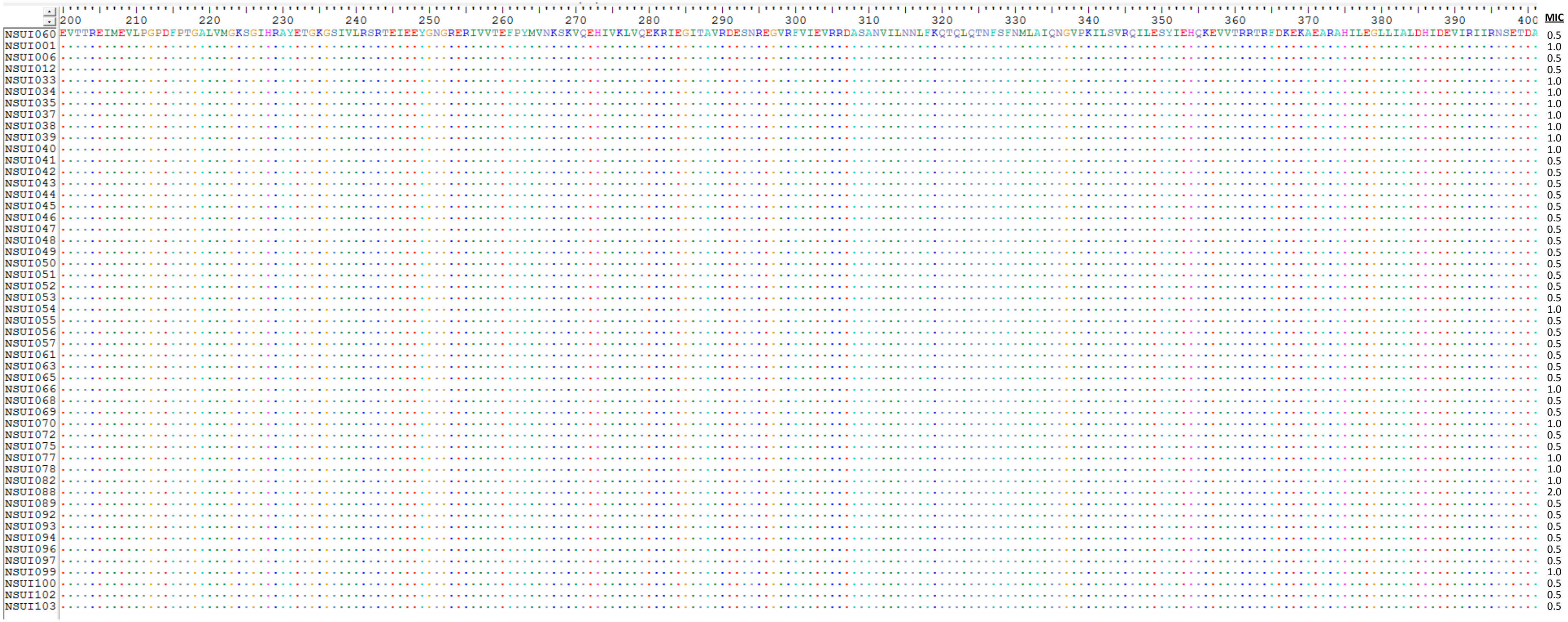

S3 Fig. Comparison of amino acid sequence of *gyrA*. The amino acid sequence for the reference strain NSUI060 is shown at the top. Dots represent conserved regions, the scale at the top represents amino acid sequence position. MICs in µg/mL to enrofloxacin for each strain are shown to the right of the figure.

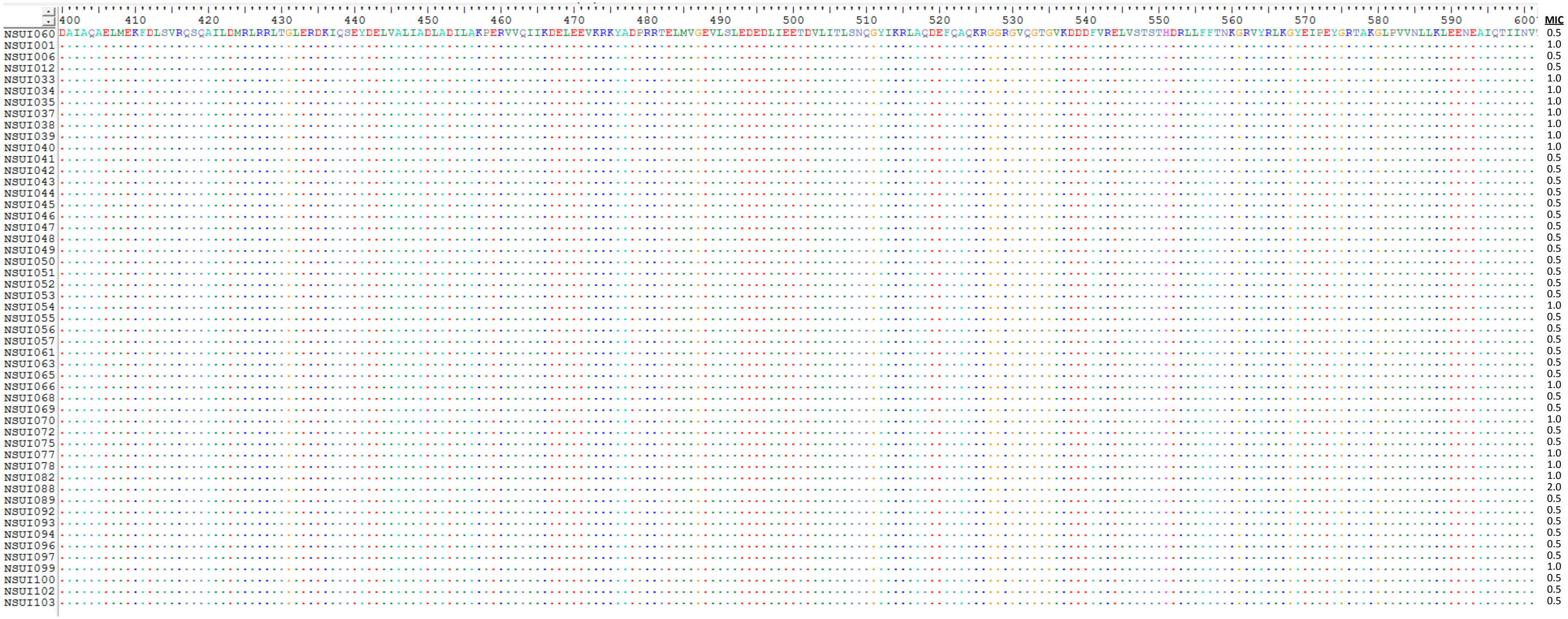

S3 Fig. Comparison of amino acid sequence of *gyrA*. The amino acid sequence for the reference strain NSUI060 is shown at the top. Dots represent conserved regions, the scale at the top represents amino acid sequence position. MICs in µg/mL to enrofloxacin for each strain are shown to the right of the figure.

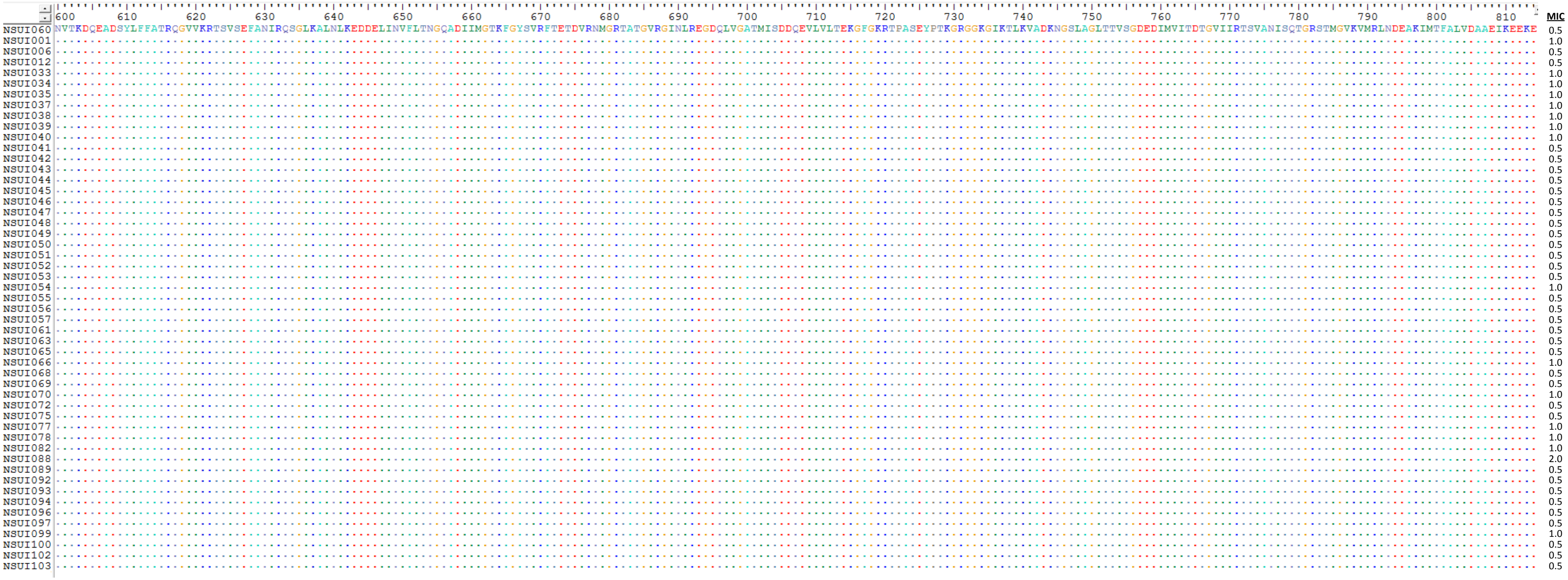

S3 Fig. Comparison of amino acid sequence of *gyrA*. The amino acid sequence for the reference strain NSUI060 is shown at the top. Dots represent conserved regions, the scale at the top represents amino acid sequence position. MICs in µg/mL to enrofloxacin for each strain are shown to the right of the figure.
