# Supplementary material for: Population Structure and Antimicrobial Resistance Profiles of Streptococcus suis Serotype 2 Sequence Type 25 Strains
Source: PLoS One. 2016 Mar 8;11(3):e0150908. doi: 10.1371/journal.pone.0150908 (PMC4783015; doi:10.1371/journal.pone.0150908)
Supplement: S5 Fig — The shades of grey depict the percent homology between elements. Integrases are depicted in dark blue, the NisK/R two-component system is depicted in green, tetrocycline resistance conferring genes are depicted in orange, lantibiotic proteins are depicted in red, and genome content is depicted in brown. (PDF) [file pone.0150908.s005.pdf]

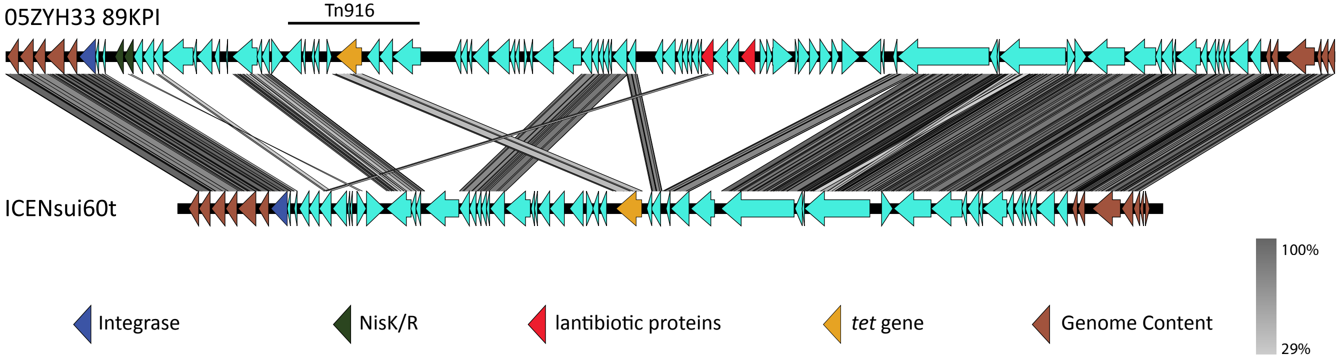

S5 Fig. Comparison of integrative and conjugative elements found in 05ZYH33 and NSUI060 containing *tet* resistance conferring genes. The shades of grey depict the percent homology between elements. Integrases are depicted in dark blue, the NisK/R two-component system is depicted in green, tetrocycline resistance conferring genes are depicted in orange, lantibiotic proteins are depicted in red, and genome content is depicted in brown.
