# Supplementary material for: Population Structure and Antimicrobial Resistance Profiles of Streptococcus suis Serotype 2 Sequence Type 25 Strains
Source: PLoS One. 2016 Mar 8;11(3):e0150908. doi: 10.1371/journal.pone.0150908 (PMC4783015; doi:10.1371/journal.pone.0150908)
Supplement: S1 Table — (PDF) [file pone.0150908.s006.pdf]

S1 Table. ***Streptococcus suis*** **strains used in this study.**

Sample	Country	Isolation Date	Host	Tissue/Disease	Illumina Sequence Data		Antimicrobial Susceptibility [c]							SRA Accession Nº
					Nº of reads	Cov [a]	ERY [D]	PEN [E]	TET [F]	AMP [G]	CEF [H]	EFX [I]	FFC [J]	
NSUI001	Canada	NA [b]	Human	NA	872,384	57.26	S	S	R	S	S	I	I	SRS1145631
NSUI006	USA	NA	Pig	Septicemia	3,253,786	144.44	R	S	R	S	S	S	I	SRS1145632
NSUI012	USA	Mar-2005	Pig	Septicemia	6,147,120	272.88	R	S	R	S	S	S	I	SRS1145638
NSUI033	Thailand	Aug-2000	Human	Meningitis	3,187,044	141.48	R	S	R	S	S	I	I	SRS1145651
NSUI034	Thailand	Sep-2000	Human	Septicemia	3,053,442	135.55	R	S	R	S	S	I	I	SRS1145658
NSUI035	Thailand	Nov-2001	Human	Meningitis	3,429,524	152.24	R	S	R	S	S	I	I	SRS1145673
NSUI037	Thailand	Oct-2002	Human	Septicemia	1,389,484	61.68	R	S	R	S	S	I	I	SRS1145671
NSUI038	Thailand	Dec-2002	Human	Septic shock	2,000,114	88.79	R	S	R	S	S	I	I	SRS1145670
NSUI039	Thailand	May-2001	Human	Septicemia	1,736,226	77.07	R	S	R	S	S	I	I	SRS1145669
NSUI040	Thailand	Mar-2002	Human	Septic shock	2,702,938	119.99	R	S	R	S	S	I	I	SRS1145668
NSUI041	Canada	Jan-2011	Pig	Brain	1,960,118	87.01	R	S	R	S	S	S	I	SRS1145592
NSUI042	Canada	Jan-2011	Pig	NA	2,438,768	108.26	R	S	R	S	S	S	I	SRS1145587
NSUI043	Canada	Feb-2011	Pig	Meningitis	1,863,292	82.71	R	S	R	S	S	S	I	SRS1145581
NSUI044	Canada	Jun-2011	Pig	Joint	3,113,672	138.22	R	S	R	S	S	S	I	SRS1145633
NSUI045	Canada	Jul-2011	Pig	Meningitis	2,739,532	121.61	R	S	R	S	S	S	I	SRS1145635
NSUI046	Canada	Aug-2011	Pig	NA	2,329,514	103.41	R	S	R	S	S	S	I	SRS1145641
NSUI047	Canada	Aug-2011	Pig	NA	1,655,206	73.48	R	S	R	S	S	S	I	SRS1145634
NSUI048	Canada	Aug-2011	Pig	Lung	4,400,548	195.35	R	S	R	S	S	S	I	SRS1145636
NSUI049	Canada	Oct-2011	Pig	Brain	925,902	41.10	R	S	R	S	S	S	I	SRS1145640
NSUI050	Canada	Nov-2011	Pig	NA	2,425,836	107.69	R	S	I	S	S	S	I	SRS1145639
NSUI051	Canada	Nov-2011	Pig	Pericardial	3,599,128	159.77	R	S	S	S	S	S	I	SRS1145637
NSUI052	Canada	Apr-2010	Pig	NA	5,912,926	262.48	R	S	R	S	S	S	I	SRS1145650
NSUI053	Canada	Apr-2010	Pig	Brain	5,131,866	227.81	R	S	R	S	S	S	I	SRS1145649
NSUI054	Canada	Jan-2007	Pig	Brain	3,310,713	217.31	R	S	R	S	S	I	I	SRS1145648
NSUI055	Canada	Feb-2007	Pig	Lung	4,404,770	289.12	R	S	R	S	S	S	I	SRS1145647
NSUI056	Canada	Jun-2007	Pig	NA	3,897,142	173.00	R	S	R	S	S	S	I	SRS1145646
NSUI057	Canada	Jan-2008	Pig	Bronchitis	2,533,078	112.45	R	S	R	S	S	S	I	SRS1145642

Sample	Country	Isolation Date	Host	Tissue/Disease	Illumina Sequence Data		Antimicrobial Susceptibility [c]							SRA Accession №
					№ of reads	Cov [a]	ERY [D]	PEN [E]	TET [F]	AMP [G]	CEF [H]	EFX [I]	FFC [J]	
NSUI060	Canada	Mar-2008	Pig	Meningitis	4,024,727	264.18	R	S	R	S	S	S	I	SRS1145643
NSUI061	Canada	Jun-2007	Pig	Lung	4,541,745	298.11	R	S	R	S	S	S	I	SRS1145644
NSUI063	Canada	Aug-2007	Pig	Meningitis	6,123,074	271.81	R	S	R	S	S	S	I	SRS1145645
NSUI065	Canada	Mar-2007	Pig	NA	3,986,122	176.95	R	S	R	S	S	S	I	SRS1145652
NSUI066	Canada	Jun-2007	Pig	Brain	6,825,802	303.00	R	S	R	S	S	I	I	SRS1145655
NSUI068	Canada	Mar-2008	Pig	Brain	4,737,434	210.30	R	S	R	S	S	S	I	SRS1145656
NSUI069	Canada	Apr-2008	Pig	Endocardium	3,952,842	175.47	R	S	R	S	S	S	I	SRS1145654
NSUI070	Canada	May-2008	Pig	Brain	4,503,350	199.91	R	S	R	S	S	I	I	SRS1145653
NSUI072	Canada	May-2008	Pig	Meningitis	5,038,722	223.67	R	S	R	S	S	S	I	SRS1145664
NSUI075	Canada	Jun-2008	Pig	Meningitis	4,024,542	178.65	R	S	R	S	S	S	I	SRS1145663
NSUI077	Canada	Jun-2008	Pig	Brain	4,717,486	209.41	R	S	R	S	S	I	I	SRS1145662
NSUI078	Canada	Jun-2008	Pig	Lung	6,849,516	304.06	R	S	R	S	S	I	I	SRS1145661
NSUI082	USA	NA	Human	NA	3,968,462	176.16	R	S	R	S	S	I	I	SRS1145660
NSUI088	Canada	Aug-2008	Pig	Multiple	7,105,960	315.44	R	S	R	S	S	R	I	SRS1145657
NSUI089	Canada	Jul-2008	Pig	Meningitis	3,298,998	146.45	R	S	R	S	S	S	I	SRS1145659
NSUI092	Thailand	Aug-2001	Human	Endocarditis	7,763,086	344.61	R	S	R	S	S	S	I	SRS1145665
NSUI093	Canada	Jan-2007	Pig	Pleura	7,353,616	326.43	R	S	R	S	S	S	I	SRS1145678
NSUI094	Canada	Feb-2008	Pig	Meningitis	8,418,242	373.69	R	S	R	S	S	S	I	SRS1145666
NSUI096	Canada	Feb-2008	Pig	Brain	7,473,816	331.77	R	S	R	S	S	S	I	SRS1145677
NSUI097	Canada	Feb-2008	Pig	Lung	7,307,112	324.37	R	S	R	S	S	S	I	SRS1145676
NSUI099	Canada	Jul-2008	Pig	Lung	6,712,044	297.95	R	S	R	S	S	I	I	SRS1145675
NSUI100	Canada	Aug-2007	Pig	Bronchitis	1,735,078	77.02	R	S	R	S	S	S	I	SRS1145667
NSUI102	Canada	Jun-2010	Pig	Meningitis	2,408,720	106.93	R	S	R	S	S	S	I	SRS1145674
NSUI103	Canada	Sep-2010	Pig	Brain	1,608,156	71.39	R	S	R	S	S	S	I	SRS1145672

[a] Cov: Coverage, based on the NSUI060 genome size of 2,255,345 bp.

[b] NA: Not Available

[c] R: Resistance based on concentrations of ≥ 1μg/mL, 2μg/mL, 2μg/mL, 2μg/mL, 8μg/mL, 2μg/mL, and 8μg/mL, respective to the order they appear in the table. S: Susceptible based on concentrations of ≤0.25μg/mL, 0.06μg/mL, 0.5μg/mL, 0.5μg/mL, 2μg/mL, 0.5μg/mL, and 2μg/mL, respective to the order they appear in the table. I: Intermediate susceptibility, strains were inhibited at a concentration between the resistant and susceptible concentrations listed above.

[D] ERY: Erythromycin

[E] PEN: Penicillin

[F] TET: Tetracycline

[G] AMP: Ampicillin

[H] CEF: Ceftiofur

[I] EFX: Enrofloxacin

[J] FFC: Florfenicol
