# Supplementary material for: Population Structure and Antimicrobial Resistance Profiles of Streptococcus suis Serotype 2 Sequence Type 25 Strains
Source: PLoS One. 2016 Mar 8;11(3):e0150908. doi: 10.1371/journal.pone.0150908 (PMC4783015; doi:10.1371/journal.pone.0150908)
Supplement: S2 Table — (PDF) [file pone.0150908.s007.pdf]

S2 Table. Characteristics of the NSUI060 genome and previously close NSUI002 and P1/7 *Streptoccocus suis* genomes.

Strain	Serotype	Sequence Type	Length	%GC content	Nº of CDSs	Genbank Acc. Nº
NSUI060	2	25	2,285,232	41.1	2,324	CP012911
NSUI002	2	28	2,255,345	41.1	2,221	CP011419
P1/7	2	1	2,007,491	41.3	1,898	NC_012925.1
